# A Pilot Study Testing the Efficacy of dCBT in Patients With Cancer Experiencing Sleep Problems

**DOI:** 10.3389/fpsyg.2022.699168

**Published:** 2022-06-08

**Authors:** Kyong-Mee Chung, Yung Jae Suh, Siyung Chin, Daesung Seo, Eun-Seung Yu, Hyun Jeong Lee, Jong-Heun Kim, Sang Wun Kim, Su-Jin Koh

**Affiliations:** ^1^Department of Psychology, Yonsei University, Seoul, South Korea; ^2^Department of Psychiatry and Behavioral Science, National Cancer Center, Goyang, South Korea; ^3^Division of Cancer Control & Policy, National Cancer Control Institute, National Cancer Center, Goyang, South Korea; ^4^Department of Obstetrics and Gynecology, Women’s Cancer Center, Yonsei University College of Medicine, Seoul, South Korea; ^5^Department of Hematology and Oncology, Ulsan University Hospital, University of Ulsan College of Medicine, Ulsan, South Korea

**Keywords:** cancer, sleep quality, mobile health, mobile device, cognitive behavioral therapy

## Abstract

**Objective:**

This pilot study aimed to evaluate the efficacy of a digital cognitive behavioral therapy (dCBT) in patients with cancer experiencing sleep problems.

**Methods:**

A total of 57 participants aged 25–65 years (6M/51F with a mean of 42.80 years and a standard deviation of 14.15 years) were randomly assigned to three groups—21 participants to a dCBT program (HARUToday Sleep), 20 participants to an app-based attentional control program (HARUCard Sleep), and 16 participants to a waitlist control group—and evaluated offline before and after the program completion. Of the 57 participants, there were a total of 45 study completers, 15 participants in each group. The dependent variables were sleep quality scores, measured by the Pittsburgh Sleep Quality Index (PSQI) and health-related quality of life scores, measured using the Short-Form 36 (SF-36), and attentional bias scores from a dot-probe computer task.

**Results:**

For both the intention-to-treat (*N* = 57) and study-completers analyses (*N* = 45, 15 for each group), a significant increase supported by a large effect size was found in the quality of sleep score of the HARUToday Sleep group compared to both the app-based attentional control and the waitlist control group. However, no significant changes were found in the quality of life and attentional bias scores.

**Conclusion:**

Our results suggest that the HARUToday Sleep app has the potential to serve as an intervention module to enhance the sleep quality of patients with cancer experiencing sleep problems.

## Introduction

Cancer is the second leading cause of death after stroke in adults worldwide (Siegel et al., 2015; World Health Organization, 2016). With recent advances in medicine, the global mortality rate of cancer declined by 22% between 1991 and 2011 (Miller et al., 2016). This decrease has changed the perspective of looking at cancer as a chronic illness for which patients should be managed over the long term for physical and psychological readjustment, even after complete recovery (Le Guen et al., 2007).

Sleep-related problems are one of the most frequently reported psychological problems experienced by patients with cancer, with 30%–60% reporting various sleep problems, such as insomnia and other extreme sleepiness, and sleep apnea (Riemann et al., 2011; Garland et al., 2014). These figures are much higher than the reported 9%–12% of physically healthy adults who experience chronic sleep-related problems (Patel et al., 2009), and the problem typically continues as a chronic condition even after the termination of cancer treatment for some patients (Palesh et al., 2012). Such a high prevalence rate is associated with cancer, which is characterized by a high mortality rate, painful and invasive treatment, and severe aftereffects (Garland et al., 2014). Such sleep-related characteristics of patients with cancer reduce immune functioning (Firth et al., 2017a) and elicit changes in carbohydrate metabolism and endocrine function (Spiegel et al., 1999). Further, they slow their recovery and exacerbate the disorder (Blask, 2009). Increased pain sensitivity (Sateia and Lang, 2008), fatigue, depression levels (Donovan and Jacobsen, 2007), and reduced quality of life (Simon and Vonkorff, 1997) also frequently arise due to sleep-related problems.

The treatment of sleep-related problems can be generally categorized into pharmacotherapy and cognitive behavioral therapy-insomnia (CBT-I; Howell et al., 2014). Although medication is the most frequently used treatment owing to its immediate effects and convenience (Asnis et al., 2016), its benefits often decrease over time and elicit severe side effects such as drug dependency, drowsiness, and confusion (Kripke et al., 2012). This is more the case for patients with cancer, as no reliable evidence of efficacy in pharmacotherapy for sleep problems has been reported (Howell et al., 2013). Conversely, growing evidence of efficacy has been accumulated in CBT-I for adults with primary insomnia (Koffel et al., 2018; van Straten et al., 2018; Boness et al., 2020), as well as diverse medical and psychiatric conditions (van Straten et al., 2018). The effectiveness of CBT-I in patients with cancer was reported in two recent reviews. Johnson et al. (2016) reviewed eight RCTs for individuals with cancer and concluded that CBT-I improved sleep efficiency, sleep latency, and wake after sleep onset with medium effect sizes (ES: *d* = 0.53, 0.43, and 0.41, respectively). Decreased insomnia severity has also been reported with a large effect size (ES: *D* = 0.77). More recently, Ma et al. (2021) reviewed 14 RCTs that tested the effectiveness of CBT-I for patients with breast cancer. They concluded that CBT-I is efficacious in reducing insomnia and improving sleep quality with medium to large effect sizes. They also reported the long-term benefits of CBT-I and demonstrated the efficacy of CBT-I regardless of the treatment delivery format (e.g., person versus remote technologies). These reviews suggest that CBT-I is a preferred treatment for sleep problems experienced by patients with cancer.

Typically, CBT-I targets first identify and modify behaviors incompatible with good sleep, followed by changing thoughts and beliefs that interfere with effective sleep (Johnson et al., 2016). To date, stimulus control, sleep restriction, relaxation, and paradoxical intention have been identified as helpful CBT-I techniques for dealing with sleep-related issues (Melton, 2018). CBT-I is the most recommended category of intervention in patients with cancer experiencing insomnia by the American Academy of Sleep Medicine (Harvey, 2010), the American College of Physicians (Qaseem et al., 2016), and the Oncology and Nursing Society (Irwin and Johnson, 2005). Despite its efficacy, the dissemination of CBT-I has been rather limited as it involves one-to-one treatment sessions, incurring notable costs concerning labor, time, and money (Beatty et al., 2016; Luik et al., 2017). CBT-I for patients with cancer is no exception, as experts in both cancer and sleeping issues are rarely found in many clinical settings (Garland et al., 2014).

Over the past decade, various digital CBT platforms have been introduced under various names, including Internet CBT (iCBT), computerized CBT (cCBT), online CBT (oCBT), and digital CBT (dCBT). As the most contemporary form of digital technology (Melton, 2018), it is created with the hope that new service delivery formats may potentially bridge the gap in feasibility, scalability, and cost-efficacy of face-to-face CBT. Since the US FDA’s approval of the reSET-O,^1^ significant efforts have been made to develop an evidence-based digital treatment with demonstrated efficacy for major mental disorders. This trend toward digitalization of treatment has also been observed in sleep disorders. In fact, a dCBT-I, called Somryst,^2^ was approved by the US FDA for sleep problems in 2020.^3^ Additionally, a few more dCBT-I^4^ have been published in the application markets. These fast movements could be possible with the demonstrated efficacy of dCBT-I for sleep issues. For example, a meta-analysis of 11 randomized controlled trials of Internet-delivered CBT targeting insomnia (eCBT-I; Zachariae et al., 2016) showed that eCBT-I programs exert significant positive effects on the severity of insomnia, sleep efficacy, and subjective sleep quality of participants, among other sleep outcomes. The study’s analysis of 1,460 participants showed that the eCBT-I improved insomnia severity, sleep efficiency, and subjective sleep quality of participants among other variables, with effect sizes (Hedges’ g) ranging from 0.21 to 1.09. More importantly, this review found that the eCBT-Is delivered comparable effects to face-to-face CBT and offered eCBT-I as an option for the treatment of insomnia. Recently, a few large-scale randomized controlled trials (RCT) have been launched (Vedaa et al., 2020) to test the efficacy of dCBT-I. The current literature, together with the need to develop a more cost-effective dCBT-I for cancer patients, points toward the viability of a dCBT-I program as a treatment for sleep-related problems in patients with cancer. However, to the best of our knowledge, no study has reported on the dCBT-I program that targets sleep-related problems experienced by patients with cancer.

Meanwhile, the measurement tools used in efficacy studies should include not only self-report measures but also objective tasks (Melton, 2018; Ham et al., 2019). For example, a dot-probe task has been recommended as a tool to assess the efficacy of Internet or mobile-based treatment programs (Ham et al., 2019). Previous studies have suggested that individuals with sleep problems show greater attentional bias to sleep stimuli compared to those with no sleep-related problems (MacMahon et al., 2006), suggesting that attentional bias is a possible objective measure of sleep. Further, many treatment outcome studies of psychological interventions have used quality of life as a secondary outcome measure, as poor sleep quality has consistently been associated with poor quality of life in past studies (Bagheri et al., 2007; Gothe et al., 2020).

Therefore, this study aimed to develop and evaluate the efficacy of an app-based CBT program targeting patients with cancer on quality of sleep, and quality of life using self-report questionnaires and a computer task. Upon findings from the previous studies, it is hypothesized that quality of sleep and quality of life will increase, while sleep-related problems would decrease after using the present dCBT program.

## Materials and Methods

### Research Participants

Participants were recruited by referrals from the oncologists in charge of three major cancer centers^5^ in South Korea or through advertisements in hospitals and public areas. These include Internet portal sites for patients with cancer, the bulletin boards of websites of cancer associations, the bulletin boards of the three hospitals, and subway advertisements.

**Figure 1:**
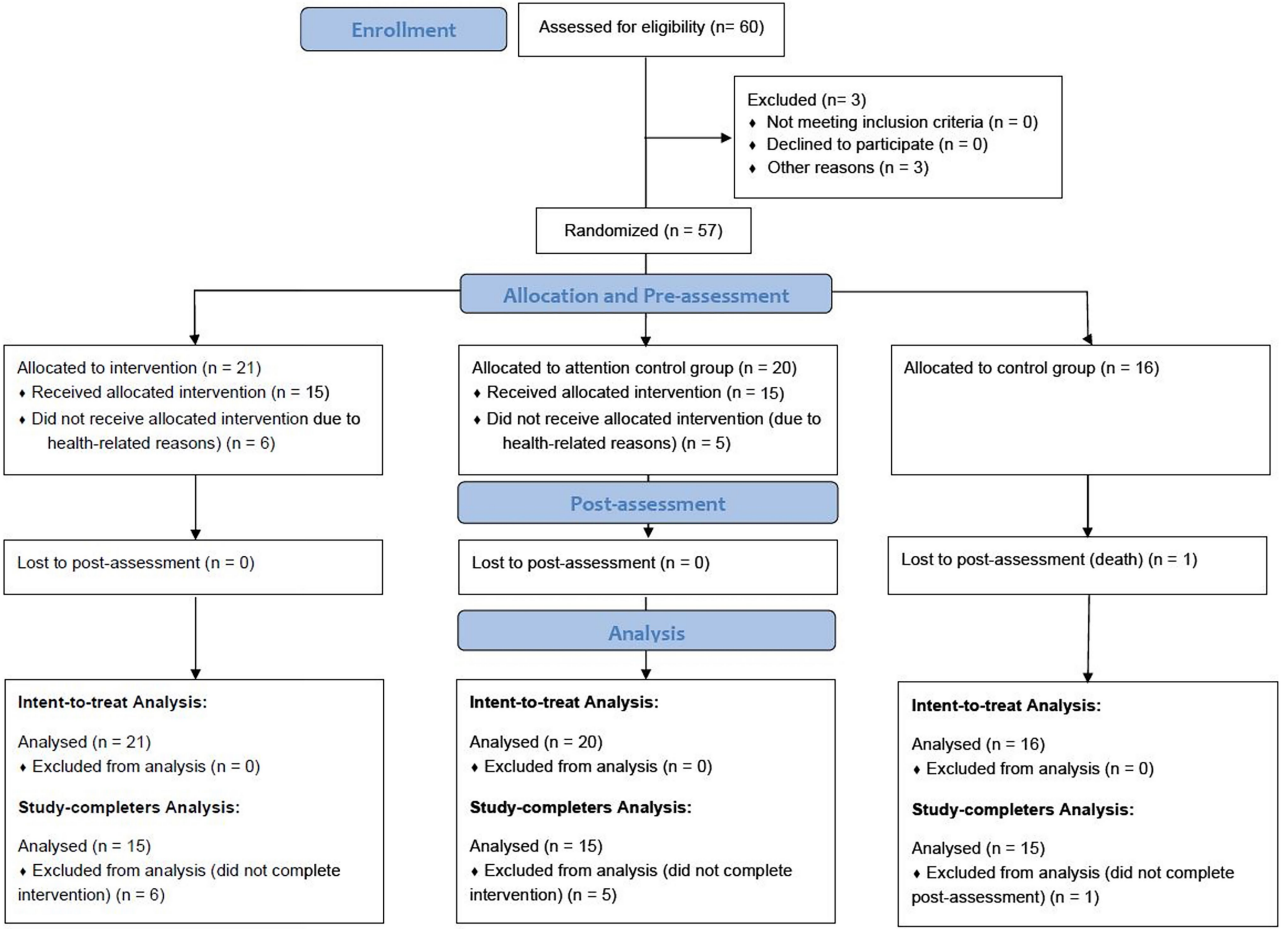
Participant flowchart.

Individuals aged 25–65 years diagnosed with any type of cancer were eligible for this study. Among the 60 patients who were recruited, 57 (six men and 51 women) met the following inclusion criteria: (1) ≥ 8.5 points on the Pittsburgh Sleep Quality Index (PSQI) and (2) no medications used (e.g., antidepressants). The exclusion criteria can be seen in Appendix. Of the 57 participants, 12 dropped out of the study due to fatigue (*n* = 5), health deterioration (*n* = 5), death (*n* = 1), or technical difficulties in using the app (*n* = 1), leaving 45 participants in total (15 per group for the three groups: an intervention group, an attention control group, and a waitlist control group). The participant flowchart is presented in Figure 1.

There were no significant differences across the three groups concerning sex, age, education level, cancer type, stage of cancer, or state of recurrence or metastasis of cancer. Sample-specific demographic information is presented in Table 1 (see Supplementary Table S1 for study completers). This study was conducted as a government-funded research project that aimed to develop and evaluate the efficacy of three app-based CBT programs.^6^ In this study, the efficacy of one of the three applications was evaluated. Although the participant recruitment methods and procedures were the same, and the three apps were launched simultaneously, outcome studies were conducted separately with different participants for each application. This study was approved by the institutional review boards of the three participating institutions (nos. 7001988-315 201901-SB-153-17, NCC-2018-0066, and UUH-2017-11-026-010). It was registered under the ISRCTN registry, a primary clinical registry, with study ID ISRCTN11480833.

**Table 1:** Participants’ demographics per group (all participants).

Group	Participants (*N* = 57)			*χ*^2^/*F*	*df*	*p*
	Intervention group (*n* = 21)	Attention control group (*n* = 20)	Waitlist control group (*n* = 16)			
**Sex (male/female)**	4/17	4/16	0/16	1.403	2	0.255
**Mean age (SD)** **range**	41.78 (11.12) 25–65	50.18 (9.97) 27–65	47.53 (10.89) 29–63	3.202	2	0.049
**Academic level, *n* (% from group)**				0.316	2	0.730
University graduate	10 (47.6)	10 (50.0)	10 (62.5)			
High school graduate	8 (39.1)	10 (50.0)	4 (25.0)			
Junior high graduate	3 (14.3)	0 (0)	2 (12.5)			
**Cancer type, *N* (% from group)**				0.531	2	0.832
Breast	9 (42.9)	7 (35.0)	10 (62.5)			
Gynecologic	4 (19.0)	3 (15.0)	2 (12.5)			
Thyroid	1 (4.8)	2 (10.0)	2 (12.5)			
Sarcoma	1 (4.8)	1 (5.0)	0 (0)			
Other	6 (28.6)	7 (35.0)	2 (12.5)			
**Stage, *n* (% from group)**				2.423	2	0.098
1st stage	7 (33.3)	5 (25.0)	6 (37.5)			
2nd stage	3 (14.3)	4 (20.0)	3 (18.8)			
3rd stage	7 (33.3)	4 (20.0)	6 (37.5)			
4th stage	2 (9.5)	4 (20.0)	1 (6.3)			
Relapse or metastasis	2 (9.5)	3 (15.0)	0 (0)			

### Measures

#### Pittsburgh Sleep Quality Index

The PSQI, developed by Buysse and colleagues, was used to measure patients’ sleep quality (Buysse et al., 1989); it was translated into and validated in Korean by Sohn et al. (2012). The 19-item PSQI is a self-report scale that screens or assesses sleep disturbance symptoms, offering seven component scores (e.g., subjective sleep quality, sleep latency, sleep duration, habitual sleep efficiency, sleep disturbances, use of sleeping medicine, and daytime dysfunction) and one composite score (e.g., global PSQI score). Each of the seven component scores has a range of 0–3, and the global PSQI score is calculated by summing up the seven component scores, which ranged from 0 to 21, with higher scores indicating a lower quality of sleep. In this study, the global PSQI score was used for the analysis. Although the classification criteria of Buysse et al. (1989) set poor sleep at five points in the global PSQI score, the cutoff point for poor sleep in this study was set to 8.5 points, as suggested by the standardization study of the PSQI in South Korea (Sohn et al., 2012). The internal consistency of the Korean version of the PSQI in the standardization study was 0.84; in this study, Cronbach’s *s* was 0.64.

#### Thirty-Six-Item Short-Form Health Survey

The SF-36 survey, developed by Ware and Sherbourne (1992) and translated into and validated in Korean by Koh et al. (1997), is used to measure subjective health-related quality of life. The SF-36 measures both physical and mental domains and includes eight subdomains. Some of the 36 items were rated on a 3-point Likert scale ranging from 1 (“very much so”) to 3 (“not at all”), whereas other items were composed of a 6-point Likert scale ranging from 1 (“for the entire month”) to 6 (“not at all”). Each item was aggregated by category, and some items were weighted according to the method suggested by Ware and Sherbourne (1992), after which the scores were converted to a 100-point scale (range = 0–100). Higher scores indicated a higher quality of life. Cronbach’s α values in the standardization study of the Korean version (Koh et al., 1997) ranged from 0.51 to 0.85; in this study, Cronbach’s *α* values ranged from 0.55 to 0.76.

#### Program Satisfaction Questionnaire

A program satisfaction questionnaire was designed for this study. Twelve questions were asked, including nine multiple-choice questions and three short-answer questions. The multiple-choice items were composed of six app component-related questions (e.g., duration of intervention, time of daily intervention, pre- and post-intervention assessments, duration of pre- and post-intervention assessments, reward system, and simplicity) and three subjective satisfaction questions (e.g., overall satisfaction, likelihood of participating again, and likelihood of recommending the program to others). These questions were also measured using a 5-point Likert scale, ranging from 1 (“very unsatisfied”) to 5 (“very satisfied”). In addition, three short-answer questions asked what participants’ favorite part about the program was, what required improvement, and what other components were required in the program, respectively.

#### Dot-Probe Task

The dot-probe task used in the study by MacLeod and Mathews (1988) was selected and modified to measure attentional bias based on the research findings that groups of patients with more severe sleep-related problems showed attentional bias toward sleep-related stimuli (Hahm and Lee, 2012; Harris et al., 2015). The participants were instructed to find the position of the dot probe following two simultaneous pictorial stimuli quickly and correctly. It was hypothesized that participants’ reaction time when the dot is presented at the same location as the picture at which participants’ attention is held would be faster than when the dot is presented at the location of the picture at which participants’ attention is not held (MacLeod and Mathews, 1988).

##### Dot-Probe Task Stimuli

In the dot-probe task, 27 positive-neutral pairs and 27 negative-neutral pairs of facial expressions conveying sleep-related stimuli were used. All stimuli were 5.5 cm × 3.7 cm, and each pictorial stimuli pair was presented side-by-side at 4.4 cm on a white background. The selection process for the stimuli used in this study is as follows.

The pictorial stimuli were searched using search terms (e.g., “sleep” and “insomnia”) and purchased from an internet site.^7^ The source of the pictorial stimuli was referenced on the last page of each task. Twenty-seven real-life pictures in which a person conveyed positive sleep-related activities (e.g., lying comfortably in a bed) were selected. This was in addition to 27 negative pictures in which a person conveyed negative sleep-related activities (e.g., unable to sleep, yawning) and 54 negative pictures in which a person was shown in a situation unrelated to sleep (e.g., working at a desk). Subsequently, 13 graduate and undergraduate students rated the stimuli for their sleep-relatedness and emotional valence levels on a 7-point Likert scale ranging from 1 (“not related to sleep at all”; negative) to 7 (“very related to sleep”; positive), following the stimuli rating procedure used by Hahm and Lee (2012). For the final pictorial stimuli, 27 positive and 27 negative sleep-related pictorial stimuli with a rating of five points or above were selected at the highest order of emotional valence level, and 54 sleep-unrelated pictorial stimuli with emotional valence levels close to the mid-range were selected.

##### Dot-Probe Task Procedure

The dot-probe task comprised two sets: positive-neutral and negative-neutral. In total, four practice trials were performed before each set to ensure that the participants understood the task. Each set comprised two blocks of 60 trials each, and the ratio in which the target stimuli appeared on the right and left, as well as the ratio in which the dot appeared on the right and left, were equal. There was 1-min resting time after each block, and the entire task, comprising two sets, lasted approximately 10 min.

**Figure 2:**
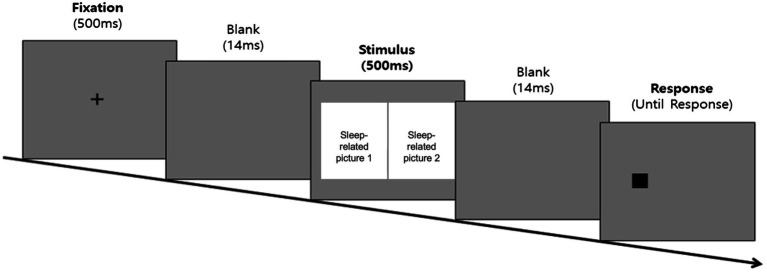
Experimental procedure diagram of the dot-probe task. The sleep-related picture stimuli was not shown in the figure due to copyright restrictions.

The task started with a fixation point (+) of 500 ms in the middle of the screen, followed by a 14 ms interstimulus interval. Subsequently, a pair of pictorial stimuli appeared on each side of the screen for 500 ms. After the stimuli disappeared, a dot (0.5 cm × 0.5 cm) randomly appeared on either side of the screen in which the pictorial stimuli had previously appeared. Participants were instructed to quickly respond to the location of the dot using a keyboard key with an attached alphabet sticker (left side = “L,” right side = “R”). The dot was presented on the screen until a response was recorded, after which the next trial started. Before the start of the task, participants completed four practice trials, followed by corrective feedback to allow them to fully understand the task. The experimental trials did not provide feedback on the given responses. The practice trials were repeated until the participant provided correct responses in at least three of the four trials. However, if the practice trials were repeated more than six times, the experiment was terminated automatically. The experimental procedure for the dot-probe task is shown in Figure 2. The experimental stimuli shown in this image were replaced with resources from Freepik.com for this study because of copyright issues.

##### Dot-Probe Task Dependent Variables

The dependent variables were the two attentional bias scores, each measured using two separate sets of stimuli—positive-neutral and negative-neutral. Attentional bias scores were calculated using the equation proposed by MacLeod and Mathews (1988). This was done by dividing the value obtained by subtracting the reaction time of the trials in which the neutral photo was located on the same side as the dot from the reaction time of the trials in which the positive or negative stimuli were located on the same side as the dot. The equation used is shown below (R = right position, L = left position, p = probe, and e = emotional stimuli). The pre- and post-intervention change scores were then calculated by subtracting the pre-intervention from the post-intervention scores.


$$Attentional\;Bias\;Score=\frac{\left(RpLe-RpRe\right)+\left(LpRe-LpLe\right)}{2}$$

### Experimental Procedure

All participants underwent screening, pre-intervention assessment, intervention in the intervention or attention control group, waiting in the waitlist control group, and post-intervention assessment.

#### Screening

Participants who agreed to enroll after receiving a brief explanation of this study completed the PSQI (Sohn et al., 2012), either online or offline. Participants who met the inclusion criteria were briefed face-to-face regarding the study, and those who gave offline written consent were randomly assigned to the three groups (HARUToday Sleep, HARUCard Sleep, or waitlist control group) after they drew a card from a shuffled deck of three cards (reflecting the three groups). After being categorized into three groups of drawn cards, participants were not informed of the group to which they were assigned.

#### Pre- and Post-intervention Assessment

Participants in all groups completed the same pre-intervention assessment package, which included two questionnaires and a dot-probe computer task. Trained and blinded research assistants administered questionnaires and computer tasks. All assessments were conducted either in the laboratory of the research team or in a designated office space within the hospitals. Within 2 weeks of completing the program, all participants visited the site and completed the post-intervention assessment and satisfaction survey. All participants received a monetary reward of approximately $110, and those in the attention control or waitlist control group were provided with the HARUToday Sleep app upon request.

#### Experimental Conditions

This study compared three groups comprising one intervention group and two control groups. The intervention group was the HARUToday Sleep group, in which the participants completed the program. The first control group was an attention control group, the HARUCard group, in which the participants could obtain sleep-related information. The purpose of the attentional control group was to control any digital placebo effects that may occur. The last group was a nontreatment control group in which the participants did not complete any type of program.

##### Intervention Group: HARUToday Sleep Group

Participants installed and logged onto (owing to privacy) the HARUToday Sleep program on their smartphones after the pre-intervention assessment and were instructed to complete one session per day for 10 weeks (66 days) at home, excluding weekends.

The HARUToday Sleep program is a modified and upgraded version of the previously developed dCBT-I (Nasi et al., 2015). This program is based on basic CBT principles, focusing on previously identified effective sleep-specific strategies, such as stimulus control, sleep restriction, relaxation, and paradoxical intention. Additionally, it teaches how to calculate sleep efficacy, follow sleep hygiene, practice lessons on the behavioral activation of the learned principles, and recognize and change dysfunctional sleep-related thoughts. The content was reviewed by patients with cancer, as well as medical and mental health professionals working in the field of oncology, *via* focus group interviews. The program illustrates examples of common sleep-related problems in which patients with cancer frequently complain and use voice narration with the minimum required reading in consideration of the average age of these patients.

**Figure 3:**
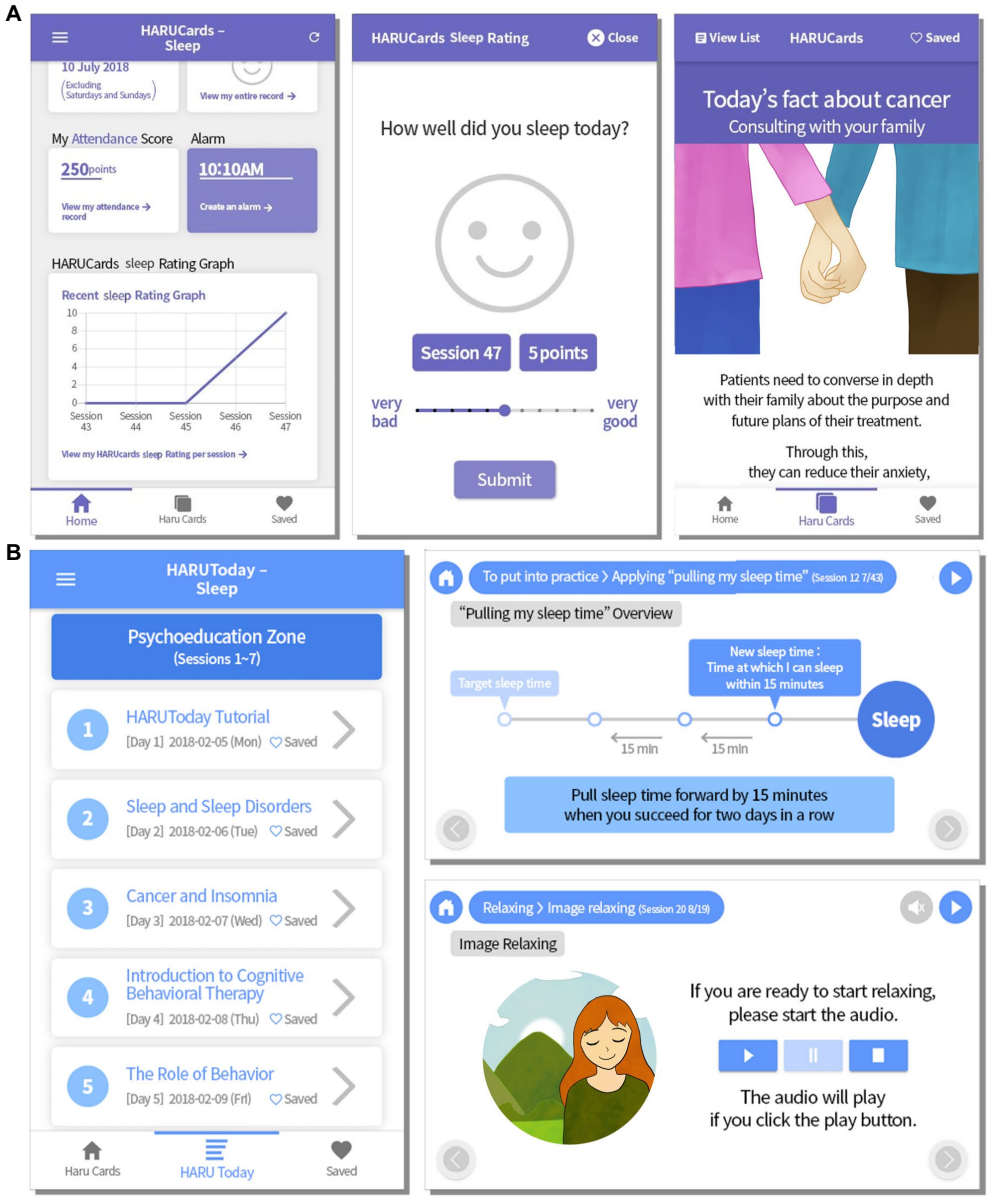
Example screen of **(A)** HARUToday Sleep and **(B)** HARUCard Sleep mobile applications.

The program comprises five zones and 48 sessions, each of which requires approximately 10–15 min to complete. The contents of the HARUToday program are listed in Table 2. All sessions included the following four phases: sleep quality rating, lessons, summaries, and quizzes. In the “sleep quality rating” phase, participants rated their sleep quality from 0 to 10 points. Other features included reward and prompting systems (Asnis et al., 2016), comprising a point system for continued attendance and completion of lessons, which was rewarded with emoji stickers, and a notification alarm to promote daily engagement. An example screen is shown in Figure 3A. The content was translated into English for this study, and the images were modified using resources from Freepik.com due to copyright issues.

**Table 2:** Content of the HARUToday program.

Zone	Sessions	Session content
Psychoeducation	Sessions 1–6	Introducing sleep-related symptoms CBT program overview Familiarization with sleep quality rating scale
Behavioral activation	Sessions 7–13	Introduction to sleep hygiene Learning how to control sleep hygiene Planning a sleep routine Conducting and evaluating the sleep routine
Relaxation training	Sessions 14–24	Introducing relaxation techniques through video and audio Introducing systematic desensitization techniques^*^
Cognitive restructuring	Sessions 25–38	Introducing the A–B–C model How thoughts can affect sleep Familiarization with how to write an A–B–C record Fixing cognitive errors that affect good sleep
Problem solving	Sessions 39–48	Learning coping strategies for anxiety-causing problems that can disturb sleep

CBT: cognitive behavioral therapy.

* Systematic desensitization, a behavioral technique by which a person is gradually exposed to an anxiety-producing event, is often used to treat sleep problems together with relaxation strategies, as sleep problems can be triggered as a result of anxiety (Sharma and Andrade, 2012).

To ensure that all participants completed the 66 days of intervention, a research assistant monitored each participant’s performance through an Internet administration page on which participants’ intervention progress was recorded. The research assistant called or sent text messages to participants who did not access the program for more than 5 days to increase their adherence to the program. Approximately 30% of the participants received a phone call or text message two to four times. Upon prompts, all participants resumed the sessions in order, as designed. The training ended for all participants when they completed all 48 sessions.

##### Attention Control Group: HARUCard Sleep Group

The HARUCard Sleep program was developed for the attention control group. This group was added to control any digital placebo effects that may arise from viewing and paying attention to a mobile application during the training period. Participants in this group received only cancer-related information or information on how to manage sleep problems for 66 days, for one session per day, excluding weekends. Each participant received the same set of cards in random order. The sleep quality ratings, as well as the reward and prompting system, were the same as in the HARUToday program (Ham et al., 2019). An example screen is shown in Figure 3B. The content was translated into English for this study, and the images were modified using resources from Freepik.com owing to copyright issues. The same login method and prompts as those used in the HARUToday program were used to increase adherence to the program in the HARUCard Sleep group.

##### Waitlist Control Group

In the waitlist control group, after completing the pre-intervention assessment, participants waited for 66 days, during which the intervention and attention control groups used the corresponding programs. There was no further contact between the participants and the researchers.

### Data Analysis

Using the G-power program, an *a priori* power analysis was conducted to determine the sample size with an error probability of 0.05, power of 0.80, and three groups. A power of 0.80 was used because a strong effect size was expected from this study, given its similarity to a previous study that explored the effects of similar CBT mobile applications targeting depression and anxiety in patients with cancer (Ham et al., 2019). Consequently, at least 66 participants were required for this study; however, only 57 participants were recruited. This lack of power is addressed in the discussion section as a study limitation.

Statistical analyses were performed using the SPSS 24.0. The analysis method was performed for both an intention-to-treat protocol (for all 57 participants, including the 12 who did not complete the intervention and post-treatment) and study completers (for the 45 participants who completed the intervention and post-treatment) using the following steps. The dependent variables were the difference scores between the pre- and post-intervention for all the outcome measures, which were normally distributed for the PSQI and SF-36 (Kolmogorov–Smirnov test > 0.05), and the attentional bias score on the dot-probe task, which were not normally distributed (Kolmogorov–Smirnov test <0.05). Additionally, no significant correlation was found in the correlational analysis between age and sleep quality; hence, data from all participants were analyzed together, regardless of age. For the intention-to-treat analysis, missing values for post-intervention were filled in using simple imputation of the baseline (pre-assessment) values, as simple imputation was the second most popular method (most popular method was to use only the complete cases for analysis) used by RCT studies with missing data (Bell et al., 2014).

To determine whether there were pre-existing differences between groups, a one-way repeated measures analysis of variance (one-way repeated measures ANOVA) was performed for the pre-intervention scores for the PSQI and SF-36, and a Kruskal–Wallis test was conducted for the pre-intervention attentional bias scores from the dot-probe task. A parametric test was used for the PSQI and SF-36 because the two were normally distributed, and a nonparametric test was used for the dot-probe task because the latter was not.

The efficacy of the HARUToday Sleep program was tested by comparing the three groups using the difference score between pre- and post-intervention using one-way repeated measures ANOVAs for the PSQI and SF-36 and a Kruskal–Wallis test for the attentional bias scores from the dot-probe task. A parametric test for the PSQI and SF-36 was used because the pre and post difference scores for these two measures were normally distributed, and a nonparametric test was used for the dot-probe task since the pre and post difference scores of the dot-probe bias were not normally distributed. Subsequently, a Bonferroni *post-hoc* test was conducted to determine the group differences, their significance, and effect sizes for any significant analysis results from the one-way repeated measures ANOVA. Further, the partial *η*^2^ was calculated to interpret as effect sizes (Cohen’s *d*; Cohen, 1988) for any significant results were calculated. The partial η^2^, and not the η^2^ was used as a measure of effect size since the two would be the same in a one-way repeated measures ANOVA analysis, and only the partial *η*^2^ has a clear benchmark criterion for small, medium, and large effect sizes for better interpretation of the results (Richardson, 2011). Lastly, the descriptive statistics for the six component-related satisfaction questions, and the three subjective satisfaction questions were calculated.

## Results

### Intention-to-Treat Analysis

#### Homogeneity Test of the Pre-intervention Scores

A one-way ANOVA test was conducted to compare the pre-intervention scores between the three groups, including all participants who completed the pre-assessment. No significant differences in the self-report questionnaires were found (PSQI: *F* = 0.25, *p* = 0.78; SF-36: *F* = 1.58, *p* = 0.21). A Kruskal–Wallis test was performed for the dot-probe tasks to compare the pre-intervention scores between the three groups, and no significant differences were found (dot-probe task-positive: *H*(2) = 1.12, *p* = 0.54; dot-probe task-negative: *H*(2) = 1.35, *p* = 0.58).

#### Effect of the Intervention

A one-way repeated measures ANOVA was performed to test whether the difference scores between pre- and post-intervention PSQI and SF-36 scores were significant. Significant differences were found across the groups before and after the intervention in the difference scores from pre-to post-intervention for PSQI (*F* = 7.76, *p* < 0.001, *ηp*^2^ = 0.38, *d* = 1.07), with a large effect size.^8^ The *post-hoc* test results showed that the PSQI scores of the intervention group declined significantly after the intervention compared with those of the experimental control group (95% CI [−12.45, −1.17], *p* < 0.01) and those of the waitlist control group (95% CI [−14.88, −2.99], *p* < 0.001). There were no significant differences between the experimental control group and the waitlist control group (95% CI [−7.94, 3.68], *p* = 1.00). Furthermore, no significant group differences were found in the difference scores for the SF-36 (*F* = 0.11, *p* = 0.89, *ηp*^2^ = 0.004; Table 3).

**Table 3:** One-way repeated measures ANOVA between the groups for the PSQI and SF-36 questionnaires (all participants).

Type	Intervention group (*n* = 21)		Attention control group (*n* = 20)		Waitlist control group (*n* = 16)		*F*	*p*	Partial *η*^2^
	Pre-*M* (*SD*)	Post-*M* (*SD*)	Pre-*M* (*SD*)	Post-*M* (*SD*)	Pre-*M* (*SD*)	Post-*M* (*SD*)			
PSQI	25.16 (4.84)	15.63 (10.00)	24.90 (4.73)	22.05 (5.26)	24.41 (6.27)	23.82 (6.09)	7.756	<0.001	0.381
SF-36	40.988 (19.529)	54.590 (19.634)	45.44 (25.37)	47.93 (26.97)	40.50 (17.08)	41.38 (16.00)	0.113	0.893	0.051

Pre, pre-assessment and post, post-assessment.

A Kruskal–Wallis test was conducted to examine whether the difference scores between pre- and post-intervention scores of the dot-probe task were significant. No attentional bias change scores for the positive and negative sleep-related stimuli in the dot-probe task [positive stimuli: H(2) = 0.89, *p* = 0.73; negative stimuli: H(2) = 0.80, *p* = 0.69] were found to be significant (Table 4).

**Table 4:** Kruskal–Wallis analysis between the groups for the dot-probe task (all participants).

Type	Intervention group (*n* = 21)		Attention control group (*n* = 20)		Waitlist control group (*n* = 16)		*χ* ^2^	*p*
	Pre-*M* (*SD*)	Post-*M* (*SD*)	Pre-*M* (*SD*)	Post-*M* (*SD*)	Pre-*M* (*SD*)	Post-*M* (*SD*)		
Positive	13.76 (13.77)	8.21 (23.36)	13.44 (8.61)	3.66 (18.22)	23.89 (19.83)	−6.42 (12.96)	0.89	0.73
Negative	5.29 (25.93)	4.76 (20.29)	4.52 (27.52)	2.12 (27.99)	5.11 (14.60)	5.12 (16.75)	0.80	0.69

Pre, pre-assessment and post, post-assessment.

### Study-Completers Analysis

#### Homogeneity Test of the Pre-intervention Scores

A one-way ANOVA was performed to compare the pre-intervention scores between the three groups of 45 participants who completed the post-assessment, which showed no significant differences in the self-report questionnaires (PSQI: *F* = 1.17, *p* = 0.32; SF-36: *F* = 1.57, *p* = 0.22). The Kruskal–Wallis test was performed for the dot-probe tasks to compare the pre-intervention scores between the three groups. No significant differences were observed between the reaction times [dot-probe task-positive: *H*(2) = 0.64, *p* = 0.73; dot-probe task-negative: *H*(2) = 0.62, *p* = 0.73].

#### Effect of the Intervention on Self-Report Questionnaires

A one-way repeated measures ANOVA was performed to test whether the difference score between pre- and post-intervention was significant across groups for PSQI and SF-36. Significant differences were found across the groups in the PSQI difference scores between pre- and post-intervention (*F* = 10.92, *p* < 0.001, *ηp*^2^ = 0.337, *d* = 1.07), with a large effect size. The *post-hoc* test results showed that the PSQI scores of the intervention group declined significantly after the intervention compared with those of the experimental control group (95% CI [0.620, 3.31], *p* < 0.010, *d* = 0.68) and the waitlist control group (95% CI [−9.20, −2.80], *p* < 0.001, *d* = 1.35). There was no significant difference between the experimental control group and the waitlist control group (95% CI [−10.14, 5.25], *p* = 1.00). No significant group differences were found in the SF-36 difference scores (*F* = 1.93, *p* = 0.16, *ηp*^2^ = 0.082; see Supplementary Table S2).

The Kruskal–Wallis test was performed to investigate the difference scores between pre- and post-intervention across groups for the attentional bias scores of positive and negative sleep-related stimuli. No significant differences were observed between the groups in attentional bias scores of positive and negative sleep-related stimuli [positive stimuli: *H*(2) = 0.64, *p* = 0.73; negative stimuli: *H*(2) = 0.62, *p* = 0.73], as presented in Supplementary Table S3.

### Participants’ Program Satisfaction

The descriptive statistics were calculated for the six component-related satisfaction questions (*M* = 3.92 and SD = 2.35) and the three subjective satisfaction questions (M = 4.12 & SD = 0.74). Of the three subjective satisfaction questions, willingness to participate once more in a similar intervention study was high (*M* = 4.33 and SD = 0.61), followed by willingness to recommend the intervention program (*M* = 4.07 and SD = 0.91). The overall question asking about whether the participant was satisfied overall had the lowest score (M = 3.97 and SD = 0.77). Of the component-related satisfaction questions, the two lowest scores were of questions which asked whether the participants were satisfied with the length of the intervention (66 days; *M* = 1.83 and SD = 0.59), and whether the participants were satisfied with the length of each lesson in the intervention (10–15 min per day). The highest satisfaction scores were on questions asking whether the mobile application was easy to use (*M* = 4.37 and SD = 0.89), followed by whether the explanation on the study before the start of the intervention was satisfactory (*M* = 4.2 and SD = 0.85), whether the reward system in the application helped in the completion of the program (*M* = 3.63 and SD = 1.13), and whether the time and place where the pre- and post- intervention took place was satisfactory (*M* = 3.43 and SD = 0.63).

## Discussion

### Main Findings

In this pilot study, a dCBT, HARUToday Sleep program was developed to decrease sleep-related problems in patients with cancer, and the effects of the app on the quality of sleep and quality of life of patients using self-report questionnaires and a computer task were investigated. The results suggested a significant increase in the quality of sleep in the HARUToday Sleep group compared with both the experimental and waitlist control groups. However, there was no significant difference in the quality of life or attentional bias between the groups.

The positive results found in the quality of sleep after intervention in this study suggest the potential of dCBT as an intervention for decreasing sleep problems among patients with cancer. The dCBT is an evidence-based program for insomnia; however, its effectiveness has been demonstrated to reduce various sleeping problems (van Straten et al., 2014; Johnson et al., 2016). This study showed the potential of dCBT as an effective program for dealing with diverse sleeping problems.

However, this is a pilot study with a small N, and no significant results were found for changes in both quality of life and attentional bias. Hence, a careful interpretation of the results is required. Further, this is the first study to investigate a newly developed dCBT, the HARUToday Sleep program, and requires additional evidence regarding its efficacy. A series of RCT with large sample sizes should be conducted to establish the efficacy of dCBT programs for patients with cancer experiencing sleep problems before its dissemination to the public.

### Research and Clinical Implications

The primary contribution of this study is that its results suggest the efficacy of the HARUToday Sleep program, dCBT, providing additional evidence for the efficacy of CBT-I in general (Nasi et al., 2015) and Internet-based CBT-I (Zachariae et al., 2016). As the HARUToday Sleep program was developed based on the CBT-I (Nasi et al., 2015), the positive results of this study suggest that CBT is an effective treatment method for sleep-related problems (Woodward, 2011). However, CBT has long been criticized for being expensive in monetary and time-related terms, resulting in limited access to treatment, where resources in terms of professionals and financial aid are scarce (Ham et al., 2019). Although studies of dCBT-I have demonstrated the efficacy of such programs in physically healthy participants with insomnia (Okajima et al., 2011), no studies have expanded on these positive results for sleep problems experienced by patients with cancer. The HARUToday Sleep program was moderately effective in improving the quality of sleep as the first dCBT to target sleep problems experienced by patients with cancer. Furthermore, this program is a self-help application program that can increase accessibility to evidence-based treatment for sleep in patients with cancer who have limited access to it (Nasi et al., 2015). The efficacy of the self-help dCBT may be a necessary entry point for the further development of more dCBT targeting the diverse symptoms of these patients.

Next, the HARUToday Sleep program has high social validity. Foremost, the overall satisfaction scores highlight that the participants were satisfied with the mobile application in general (total average = 81.45%). The other two highest categories that also scored >80% were “willingness to recommend the app” and “willingness to re-participate.” Second, previous treatment outcome studies testing app-based interventions had a mean patient age range of 18–47 years, and they more frequently targeted teens and patients in their 30s (Crabb et al., 2012). Few studies have been conducted on participants in their 40s, and a positive correlation has been reported between age and dropout rate in technology-based interventions (Firth et al., 2017b). The mean participant age in this study was 44.37 years, and the dropout rate was 21%, which is below the rate observed in most previous studies (Firth et al., 2017b). Of the participants who dropped out, only one participant dropped out owing to technical difficulties related to the app. The rest dropped out because of health-related reasons. Third, the comparably low dropout rate of participants throughout the 66 days of continuous training indicates that the participants found the application to be helpful at the least and that there were no major technical difficulties in using the program daily. This indicates that the self-help CBT mobile application developed in this study has the potential for wide use in the actual mobile application market.

Although the factors resulting in such a low dropout rate are unclear, physicians’ involvement, accessibility to psychological services, and monetary compensation of $110 to complete the post-assessment may have provided higher motivation to complete the program. Most participants were recruited by their attending physicians at the hospital where they received cancer treatment. This may have increased the patients’ perceived trustworthiness of the app. Moreover, this app provided free and easily accessible psychological services, which is a rare opportunity for this population in South Korea. Finally, as only those who completed the intervention were asked to participate in the post-assessment, monetary compensation could have been a motivator for them to complete the post-assessment. However, these are speculations, and further studies should focus on the possible attributing factors of the present app-based intervention on participants’ dropout rates.

No significant change in the quality of life after the intervention was unexpected, as previous studies have reported that sleep quality affects quality of life in general (Bagheri et al., 2007). However, some speculations can be made. First, it may have been due to the small sample size, which may have created a large variance in the sampling distribution (Wickenberg-Bolin et al., 2006). Second, it could have been related to the physical health condition of participants in this study. Some of them were under cancer treatment, suffered from aftereffects of the vigorous cancer treatment, or were at the recovery stage with a weaker health condition, or changes in sleep quality of participants may not have been sufficient to generalize to other areas, including quality of life. Another possibility is that the directionality of the effects is opposite: quality of life needs to be increased for sleep quality to increase or sleep-related problems to reside. Finally, the 66 days of intervention may not have been sufficient for patients with cancer to habituate the sleep hygiene methods introduced in the program, especially when the CBT methods had to be self-implemented. Therefore, the methods by which to increase the quality of life of patients with cancer should be considered in future studies.

### Limitations

This study has several limitations. First, it is a pilot study with an underpowered sample size. Recruiting patients with cancer who have sleeping problems was extremely difficult in this study, as medication is a typical and more acceptable choice of treatment for this population, at least in South Korea. Replication is a must with a large sample size, for which strategies to facilitate the recruitment procedure should be well planned. Second, this pilot study used the PSQI as a primary outcome measure and did not include diagnostic tools such as the Insomnia Severity Index. The PSQI is a subjective measure for sleep quality. This is the primary reason for not categorizing the HARUSleep program as a dCBT-I. To establish the efficacy of the HARUSleep program as a dCBT-I for cancer patients, subsequent studies should include diagnostic tools for sleep. Third, future studies should also consider the effects of age and other psychological and cancer-related factors (e.g., depression, anxiety, type of cancer, aftereffects, etc.) on sleep quality, as a positive correlation between these variables and sleep has been reported in a few previous studies (Donovan and Jacobsen, 2007; Melton, 2018). These variables were not included in this study due to its focus on preliminary efficacy as a pilot study and because of the small sample size. Further, subsequent studies would benefit from measuring more primary outcome measures, as sleep quality can be measured by different hallmark variables, such as sleep duration or inability to fall asleep. Finally, the lasting effects of dCBT should be evaluated by including a long-term follow-up assessment, which is critical to establish the efficacy of any psychological treatment.

### Conclusion

This study was conducted to examine the preliminary efficacy of dCBT in patients with cancer experiencing sleep problems. The HARUToday Sleep program was developed, and the results suggested that the program has the potential to decrease sleep problems in cancer patients. However, this is a pilot study with a small sample size; hence, careful interpretation should be made. Nevertheless, this study has strengths in terms of experimental design—it was an RCT and included a digital-placebo group. The improved quality of sleep after receiving the HARUToday Sleep program suggested that this new program has the potential to become an efficacious program to resolve sleep problems among patients with cancer. Again, this is the first step in establishing the efficacy of dCBT for cancer patients and requires a series of RCTs with larger sample sizes. Through these studies, mediating factors such as demographic, psychological, and cancer-related variables (age, depression, anxiety, cancer type, aftereffects, etc.) related to the efficacy of this program should be identified to better target those patients who will benefit from the program before the dissemination of the program to clinical settings. Future studies should aim to discover the underlying mechanism (e.g., change in biased thinking, decrease in negative thinking, etc.) of efficacious dCBT and the involved technical processes (e.g., components of the program, delivery method of the program, etc.). Finally, the results of the present study may possibly lay the ground to determine the efficacy of dCBT programs for sleep in terminally ill patients with chronic diseases.

## Data Availability Statement

The raw data supporting the conclusions of this article will be made available by the authors, without undue reservation.

## Ethics Statement

This study was carried out under the approval of the Institutional Review Boards (IRBs) of Yonsei University, the National Cancer Center, and Ulsan University Hospital in South Korea. All subjects provided written informed consent.

## Author Contributions

K-MC developed the theoretical framework, designed the experiments, and directed the project. YS designed the experiments, collected the data, designed and built the computer task, analyzed the data, and wrote the manuscript. SC collected the data, built the computer task, analyzed the data, and wrote the manuscript. E-SY, HL, J-HK, SK, DS, and S-JK recruited the participants, collected the data, and wrote the manuscript. All authors contributed to the article and approved the submitted version.

## Funding

This study was supported by a grant from the National R&D Program for Cancer Control, Ministry of Health and Welfare, Republic of Korea (HA16C0021).

## Conflict of Interest

The authors declare that the research was conducted in the absence of any commercial or financial relationships that could be construed as a potential conflict of interest.

## Publisher’s Note

All claims expressed in this article are solely those of the authors and do not necessarily represent those of their affiliated organizations, or those of the publisher, the editors and the reviewers. Any product that may be evaluated in this article, or claim that may be made by its manufacturer, is not guaranteed or endorsed by the publisher.

## Supplementary Material

The Supplementary Material for this article can be found online at: https://www.frontiersin.org/articles/10.3389/fpsyg.2022.699168/full#supplementary-material

## Footnotes

^1^An application-based cognitive behavior program (CBT) for patients with substance use disorder. Website link: https://peartherapeutics.com/products/reset-reset-o/

^2^Somryst, https://peartherapeutics.com/products/somryst/

^3^ https://www.mobihealthnews.com/news/fda-clears-pears-digital-insomnia-therapeutic-through-510k-pre-cert-pilot-pathways

^4^Sleepio, https://www.bighealth.com/sleepio/; CBT-i Coach, https://mobile.va.gov/app/cbt-i-coach; Clicadian, https://www.clicktherapeutics.com/products/

^5^National Cancer Center in South Korea, Severance Medical Center at Yonsei University, and Ulsan University Medical Center. The first two are located in Seoul, South Korea, and the third hospital is located in Ulsan, Kyongsangnamdo, South Korea.

^6^This study was supported by a grant from the National R&D Program for Cancer Control, Ministry of Health and Welfare, South Korea (HA16C0021).

^7^ www.shutterstock.com

^8^Effect sizes were interpreted as small (*d* = 0.2), medium (*d* = 0.5), or large (*d* = 0.8) based on benchmarks suggested by Cohen (1988).

## Appendix

### A. Inclusion and exclusion criteria

Inclusion criteria:

- All genders.
- Age range of 25–65 years.
- Has a past diagnosis of any type of cancer.
- 8.5 points or more on the Pittsburgh Sleep Quality Index (PSQI).

Exclusion criteria

- Taking prescribed medication (such as antidepressants).
- Taking sleep-related medication.
