# Supplementary material for: A Pilot Study Testing the Efficacy of dCBT in Patients With Cancer Experiencing Sleep Problems
Source: Front Psychol. 2022 Jun 8;13:699168. doi: 10.3389/fpsyg.2022.699168 (PMC9216201; doi:10.3389/fpsyg.2022.699168)
Supplement: Supplementary file 1 [file Data_Sheet_1.docx]

Supplementary Information

TABLE S1. Participants’ demographics per group (Study completers)

| Group | | Participants (N = 45) | | | | | | |  | |  | | |  | |  |  |  |
| --- | --- | --- | --- | --- | --- | --- | --- | --- | --- | --- | --- | --- | --- | --- | --- | --- | --- | --- |
|  |  | Intervention Group  (n = 15) | | Attention Control Group  (n = 15) | | Waitlist Control  Group  (n = 15) | | | *χ^2^/F* | | *df* | | | *p* | |  |  |  |
| Sex (male/female) | | 2/13 | | 1/14 | | 0/15 | | | 1.182 | | 2 | | | .554 | |  |  |  |
| Mean age (*SD*), range | | 44.37  (11.86)  25–65 | | 48.72  (10.91)  29–63 | | 46.63 (10.38)  27–64 | | | .462 | | 2 | | | .634 | |  |  |  |
| Academic level, n(% from group) | | | | | | | | | 5.280 | | 2 | | | .260 | |  |  |  |
| University graduate | | 9 (61.2) | | 10 (66.6) | | 10 (66.6) | | |  | |  | | |  | |  |  |  |
| High school graduate | | 5 (33.2) | | 5 (33.4) | | 3 (20.1) | | |  | |  | | |  | |  |  |  |
| Junior high graduate | | 1 (5.6) | | 0 (0) | | 2 (13.3) | | |  | |  | | |  | |  |  |  |
| Cancer type,  N (% from group) | |  | |  | |  | | | 5.588 | | 2 | | | .693 | |  |  |  |
| Breast | | 8 (53.3) | | 6 (40.0) | | 9 (60.0) | | |  | |  | | |  | |  |  |  |
| Gynecologic | | 2 (13.4) | | 2 (13.4) | | 2 (13.4) | | |  | |  | | |  | |  |  |  |
| Thyroid | | 1 (6.7) | | 2 (13.4) | | 2 (13.3) | | |  | |  | | |  | |  |  |  |
| Sarcoma | | 1 (6.7) | | 1 (6.7) | | 0 (0) | | |  | |  | | |  | |  |  |  |
| Other | | 3 (19.9) | | 4 (26.5) | | 2 (13.3) | | |  | |  | | |  | |  |  |  |
| Stage, n (% from group) | | | | | | | | | 6.873 | | | | 2 | | | .333 |  |  |
| 1st stage | | 5 (33.3) | | 5 (33.3) | | 6 (40.0) | |  | | |  | | |  | |  |  |  |
| 2nd stage | | 5 (33.3) | | 2 (13.4) | | 3 (20.0) | |  | | |  | | |  | |  |  |  |
| 3rd stage | | 3 (20.0) | | 5 (33.3) | | 5 (33.3) | |  | | |  | | |  | |  |  |  |
| 4th stage | | 2 (13.4) | | 3 (20.0) | | 1 (6.7) | |  | | |  | | |  | |  |  |  |
| Relapse  or metastasis | | 1 (6.7) | | 3 (20.0) | | 0 (0) | |  | | |  | | |  | |  | | |
|  | | | | | | | |  | | |  | | |  | |  | | |
|  | |  | |  | |  | |  | | |  | |  | | |  | | |
|  | |  | |  | |  | |  | | |  | |  | | |  | | |
|  | |  | |  | |  | |  | | |  | |  | | |  | | |

TABLE S2. One-way repeated measures ANOVA between the groups for the PSQI and SF-36 questionnaires (Study completers)

| Type | Intervention Group  (n = 15) | | Attention Control Group  (n = 15) | | Waitlist Control Group  (n = 15) | | *F* | *p* | Partial η^2^ |
| --- | --- | --- | --- | --- | --- | --- | --- | --- | --- |
|  | Pre-  *M* (*SD*) | Post-  *M* (*SD*) | Pre-  *M* (*SD*) | Post-  *M* (*SD*) | Pre-  *M* (*SD*) | Post-  *M* (*SD*) |  |  |  |
| PSQI | 14.93 (3.369) | 8.47 (3.314) | 14.73 (3.348) | 11.27 (3.973) | 13.27 (3.035) | 12.80 (3.028) | 10.921 | < .001 | .337 |
| SF-36 | 40.988 (19.529) | 54.590 (19.634) | 40.009 (21.192) | 46.361 (19.546) | 51.351 (17.345) | 46.815 (14.436) | 1.931 | .158 | .082 |

Note: pre = pre-assessment; post = post-assessment

TABLE S3. Kruskal-Wallis analysis between the groups for the dot-probe task (Study Completers)

| Type | Intervention Group  (n = 21) | | | Attention Control Group  (n = 20) | | Waitlist Control Group  (n = 16) | |  | |  |  |
| --- | --- | --- | --- | --- | --- | --- | --- | --- | --- | --- | --- |
|  | Pre-  *M* (*SD*) | | Post-  *M* (*SD*) | Pre-  *M* (*SD*) | Post-  *M* (*SD*) | Pre-  *M* (*SD*) | Post-  *M* (*SD*) | | *X^2^* | *p* |  |
| Positive | 16.16  (14.95) | 25.49  (25.10) | | 12.87  (9.23) | 16.05  (16.34) | 26.43  (24.62) | 20.21  (13.69) | .64 | | .73 |  |
| Negative | 8.30  (21.38) | 5.63  (29.26) | | 3.81  (17.30) | 4.89  (30.13) | 5.23  (23.31) | 5.39  (19.73) | .62 | | .73 |  |

Note: pre = pre-assessment; post = post-assessment
